# Hydrogeochemical changes before and during the 2016 Amatrice-Norcia seismic sequence (central Italy)

**DOI:** 10.1038/s41598-017-11990-8

**Published:** 2017-09-15

**Authors:** Marino Domenico Barberio, Maurizio Barbieri, Andrea Billi, Carlo Doglioni, Marco Petitta

**Affiliations:** 1 Dipartimento di Scienze della Terra, Sapienza University of Rome, Rome, Italy; 2 Consiglio Nazionale delle Ricerche, IGAG, Rome, Italy; 3 Istituto Nazionale di Geofisica e Vulcanologia, Rome, Italy

## Abstract

Seismic precursors are an as yet unattained frontier in earthquake studies. With the aim of making a step towards this frontier, we present a hydrogeochemical dataset associated with the 2016 Amatrice-Norcia seismic sequence (central Apennines, Italy), developed from August 24^th^, with an M_w_ 6.0 event, and culminating on October 30^th^, with an M_w_ 6.5 mainshock. The seismic sequence occurred during a seasonal depletion of hydrostructures, and the four strongest earthquakes (M_w_ ≥ 5.5) generated an abrupt uplift of the water level, recorded up to 100 km away from the mainshock area. Monitoring a set of selected springs in the central Apennines, a few hydrogeochemical anomalies were observed months before the onset of the seismic swarm, including a variation of pH values and an increase of As, V, and Fe concentrations. Cr concentrations increased immediately after the onset of the seismic sequence. On November 2016, these elements recovered to their usual low concentrations. We interpret these geochemical anomalies as reliable seismic precursors for a dilational tectonic setting.

## Introduction

Earthquake forecasting is the holy grail for most, if not all, geoscientists^1,2^. Major progress has been made using changes and anomalies in foreshock sequences^3^, V_p_/V_s_^4–7^, electric and magnetic fields^8^, gas emissions^9^, surface deformations^10–15^, groundwater levels^16–20^, and other features^2,21,22^. However, the main goal, i.e. the short-term forecasting of earthquakes, remains elusive and largely unattained. An effective solution for such a huge issue might be found, in the future, in systematic measurements with multi-parametric networks operating over large regions and over the long term. Owing to influx from deep crustal fluids in active tectonic areas, groundwater monitoring is considered a fundamental tool for investigating pre-seismic signals of rocks undergoing accelerated strain^23–31^. Promising examples are from Iceland, where, four to six months before two consecutive extensional-transtensional fault earthquakes (M_w_ 5.5 and 5.6 on October 2012 and April 2013 respectively), the chemistry of the groundwater sampled about 75 km from the earthquake epicentres changed, likely in relation to pre-seismic crustal dilation^30,31^. In 2002, in the same study area in Iceland, similar hydrogeochemical changes had already been recorded one to ten weeks before an M_w_ 5.8 transtensional fault earthquake, at a distance of about 90 km from the epicentre^26^.

**Figure 1:**
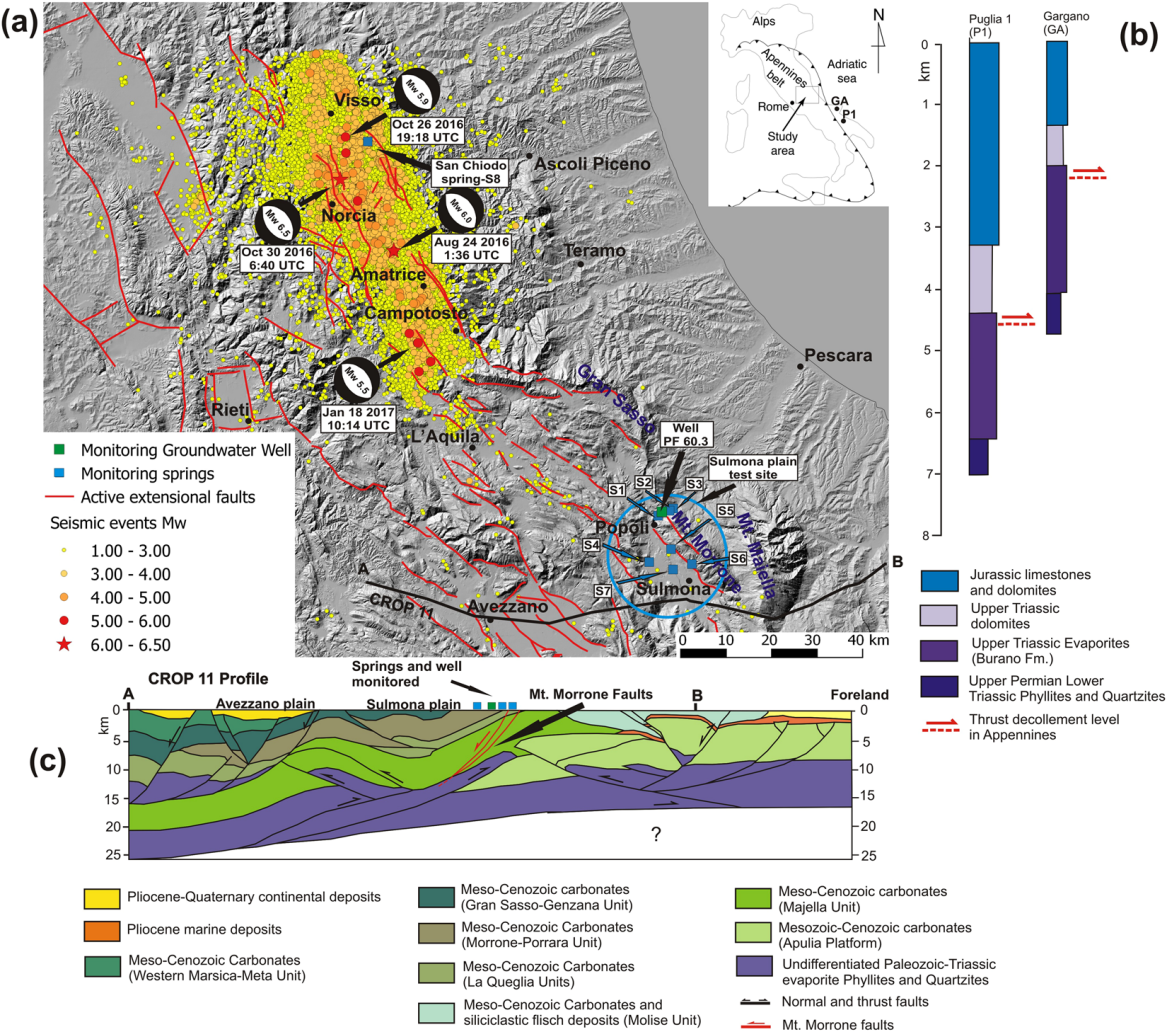
Geological setting. This figure has been drawn using CorelDRAW^97^. (**a**) Map of central Apennines (see location in upper right inset) showing earthquake epicentres of the 2016–2017 sequence. Seismic data (including focal mechanisms) are from the INGV database (available online at http://cnt.rm.ingv.it/) between April 1^st^, 2016, and March 30^th^, 2017 (M_w_ ≥ 1.0) (Supplementary Table S1). Active faults (all extensional) are from the Ithaca database (available online at http://www.isprambiente.gov.it/en/projects/soil-and-territory/italy-hazards-from-capable-faulting). Base digital elevation model is from the ISPRA database SINAnet (available online at http://www.sinanet.isprambiente.it/it). Location of the well (PF 60.3) and springs monitored and analyzed in this work (S1-S8 in the Sulmona Plain Test Site and San Chiodo area) are displayed with blue and green squares, respectively (Supplementary Table S2). (**b**) Simplified stratigraphic logs from the Gargano (GA) and Puglia 1 (P1) deep wells drilled in the Apulian-Adriatic foreland of the Apennines fold-thrust belt. See well location in upper right inset of (**a**). Data are from the Videpi database (available online at http://unmig.sviluppoeconomico.gov.it/videpi/pozzi/pozzi.asp; see also ref.^41^). (**c**) Interpretation of the CROP-11 deep seismic profile (modified after ref.^41^). See A-B profile track in (**a**). Note the location and depth of the extensional Mt. Morrone Fault (red faults) that is adjacent to the well and springs considered in this paper (Sulmona Plain Test Site, SPTS).

Following these examples, intermittently since July 2014 and systematically since January 2016, we have hydrogeologically and hydrogeochemically monitored a 100 m deep well (PF 60.3) and a group of springs in the Sulmona Plain Test Site (SPTS), central Apennines, Italy (Fig. 1a), where active extensional faults and related seismicity occur^32–35^.

On August 24^th^, 2016, a seismic sequence started in the extensional area of the central Apennines. At the end of June 2017, the sequence had included more than 70,000 earthquakes. Most earthquakes were normal faulting events, nine of which with M_w_ ≥ 5. The strongest events were the initial M_w_ 6.0 earthquake (August 24^th^) and the M_w_ 6.5 mainshock (October 30^th^), located, respectively (Fig. 1a), between about 70 and 90 km north-northwest of the SPTS^36–38^. The August 24^th^ event, in particular, killed 294 people in the Amatrice area. In addition to significant post-mainshock changes in groundwater levels and spring discharges, we recorded, before the onset of the sequence, some hydrogeochemical changes that could potentially represent earthquake precursors.

## Geological Setting

The central Apennines (Fig. 1) is a NW-SE-trending segment of the Apennines accretionary prism, developed during Oligocene-Quaternary times, with a main eastward vergence and a classical forelandward propagation of thrust sheets, followed by backarc extension cross-cutting the previously-formed orogenic prism^39–42^. The thrust sheets are mainly composed by the Meso-Cenozoic sedimentary succession pertaining to the Apulian-Adriatic plate margin that is still westerly-subducting beneath the Apennines. This succession is known from deep wells drilled in the Apulian-Adriatic foreland and includes, from top to bottom: (1) Meso-Cenozoic limestones and dolostones; (2) Triassic evaporites with anhydrites and dolomites; and (3) Permian-Triassic phyllites and quartzites (Fig. 1b). This Apulian-Adriatic succession is duplicated and eastwardly-imbricate in the Apennines fold-thrust belt, where it is also topped by syn- and post-orogenic siliciclastic marine and continental deposits^41^ (Fig. 1c). In the Apennines, both the Cenozoic clayey syn-orogenic deposits and the Triassic anhydritic evaporites are known as important sealing levels for groundwater, hydrocarbons, and deep endogenic gases, such as CO_2_^43–47^.

In the study area, the deep architecture of the Apennines belt is known from the CROP-11 deep seismic profile^41^, which shows a series of E-verging thrusts and a few W-verging back-thrusts, involving the Adriatic succession of sedimentary rocks down to the Paleozoic units. The sole thrust of the Apennines occurs at more than 20 km in the inner sector (west), shallowing to about 10 km toward the foreland (east; Fig. 1c).

In the central Apennines, since Miocene time, contraction to the east and backarc extension to the west have eastward migrated as a result of the easterly retreat of the westerly-directed subduction. Compression is still active in the foreland to the east, whereas the Apennines have undergone extension along the ridge and to the west of it^48–51^. This extensional regime is, at present, particularly seismogenic along the axis of the Apennines^32^. Between late Miocene and present times, during the eastward transit of the backarc extensional tectonic wave, the accretionary prism has been dissected and down-faulted by a system of NW-striking extensional faults (Fig. 1a), which bound several intramountain basins filled by Plio-Quaternary continental deposits^52^. The collapse of the upper brittle crustal layer in the Apennines has been interpreted as the main source of seismicity in the area, with gravity energy being the main propellant^53^. Normal faults have generated historical and instrumental seismicity of up to M_w_ 7.0 earthquakes, e.g., the Avezzano, 1915 (M_w_ 7.0)^54^, L’Aquila, 2009 (M_w_ 6.3)^55^, and Amatrice-Norcia, 2016 (M_w_ 6.0 and 6.5) earthquakes^56^.

In this region, most strong earthquakes (M_w_ > 6.0) nucleate at depths of 8–10 km, and the co-seismic slip propagates upward along carbonate-hosted faults. One of these active extensional faults is the Mt. Morrone Fault, striking NW-SE by about 25 km and dipping toward SW by about 50–60° (Fig. 1a,c). The SPTS (Supplementary Table S2) is adjacent to the Mt. Morrone Fault, with springs located both on the fault footwall and on the fault hanging wall (Fig. 1a,c). This fault is considered as potentially responsible for past earthquakes of up to M_w_ 6.5 or even stronger, and its last activation probably occurred during the 2^nd^ Century AD^35,57–59^. The Mt. Morrone Fault is imaged in the CROP-11 deep seismic profile down to depths of about 12 km, cutting through the sedimentary carbonate and evaporitic cover down to the Paleozoic phyllites^41^ (Fig. 1c). The Mt. Morrone Fault, together with the normal faults activated during the 2009 L’Aquila and 2016 Amatrice-Norcia earthquakes, belongs to the array of seismically-active NW-striking normal faults of the central Apennines^32,60–62^.

The 2016–2017 seismic sequence of central Apennines started on August 24^th^, with an M_w_ 6.0 earthquake in the Amatrice area (hypocentre depth ~8 km). The sequence occurred in the gap between the 1997–98 Colfiorito (M_w_ 5.4 and M_w_ 5.9 earthquakes) and the 2009 L’Aquila (Mw 6.3 earthquake) seismic sequences. In addition to the above-mentioned M_w_ 6.0 Amatrice and M_w_ 6.5 Norcia earthquakes, the sequence has included, until the time of writing (July, 2017), seven further main earthquakes, with an M_w_ of between 5.0 and 5.9 (Supplementary Table S1; Fig. 1a). In particular, on January 18^th^, 2017, the sequence migrated a few kilometres southward, where an M_w_ 5.5 earthquake was accompanied within a few hours by three further M_w_ > 5 earthquakes (one M_w_ 5.4 and two M_w_ 5.1 earthquakes) in the Amatrice-Campotosto area (Fig. 1a), with hypocentres located at ~9 km depth (Supplementary Table S1). The sequence has activated a set of NW-striking normal faults for a total length of about 70 km (Fig. 1a). The maximum coseismic slip seismologically-determined for the M_w_ 6.0 and 6.5 earthquakes was ~1 and ~3 m, respectively^36–38,63^.

## Hydrogeological Setting and Site Selection

The central Apennines belt is characterised by huge fractured aquifers hosted by Meso-Cenozoic carbonate sequences (Fig. 1b,c), often compartmentalised and sealed by clay-rich low-permeability layers (aquicludes), such as syn-orogenic siliciclastic marine deposits (Fig. 1c). The post-orogenic extensional tectonics have contributed to producing thick Plio-Pleistocene continental deposits filling the intramountain basins and river valleys, thus forming aquitards for the underlying carbonate aquifers^64,65^. The recharge of the carbonate aquifers is very high (up to 1000 mm/a, 70–80% of rainfall), driven mostly by fractures and secondarily by dissolution features^66,67^. Most springs drain the aquifers with very high discharge rates (0.5–1 m^3^/s on average, up to 18 m^3^/s) and steady regimes, where a mild recharge period in winter-spring time is usually followed by a depletion phase in summer-autumn time^64,68^.

The study area (i.e. SPTS, Fig. 1a) is located between the Gran Sasso and Mt. Morrone carbonate aquifers, where groundwater flows converge to feed springs with discharges between less than 0.01 m^3^/s and more than 1 m^3^/s. Carbonate aquifers host regional groundwater flow and feed springs located at ridge piedmonts, the latter of which frequently correspond to active faults. Consequently, the predominant water type from these springs is calcium bicarbonate, occasionally affected by deep mixed sulphate-calcium-bicarbonate waters. Contribution by deep fluids is enhanced by active faulting and is superimposed on the regional flow of carbonate aquifers^66,69^.

To monitor groundwater flow at the boundary of the Mt. Morrone ridge in the SPTS, we selected the monitoring well PF 60.3, which draws out from the carbonate aquifer lying under recent alluvial deposits^70–73^. For hydrogeochemical and stable isotope monitoring we then selected seven basal springs fed by the regional flow from the same carbonate aquifers. In January 2016, we started our hydrogeochemical monitoring on four of these springs (S1, S2, S3, and S4; Supplementary Tables S2 and S3), which are characterised by the highest temperature and electrical conductivity among the known springs in the area, evidencing a deep fluid contribution including CO_2_ and H_2_S^66^. Starting from February 2016, we added three additional springs to the monitoring network (S5, S6 and S7; Supplementary Tables S2 and S3), which are directly fed by regional flow in carbonate aquifers with no evidence of deep fluids.

**Figure 2:**
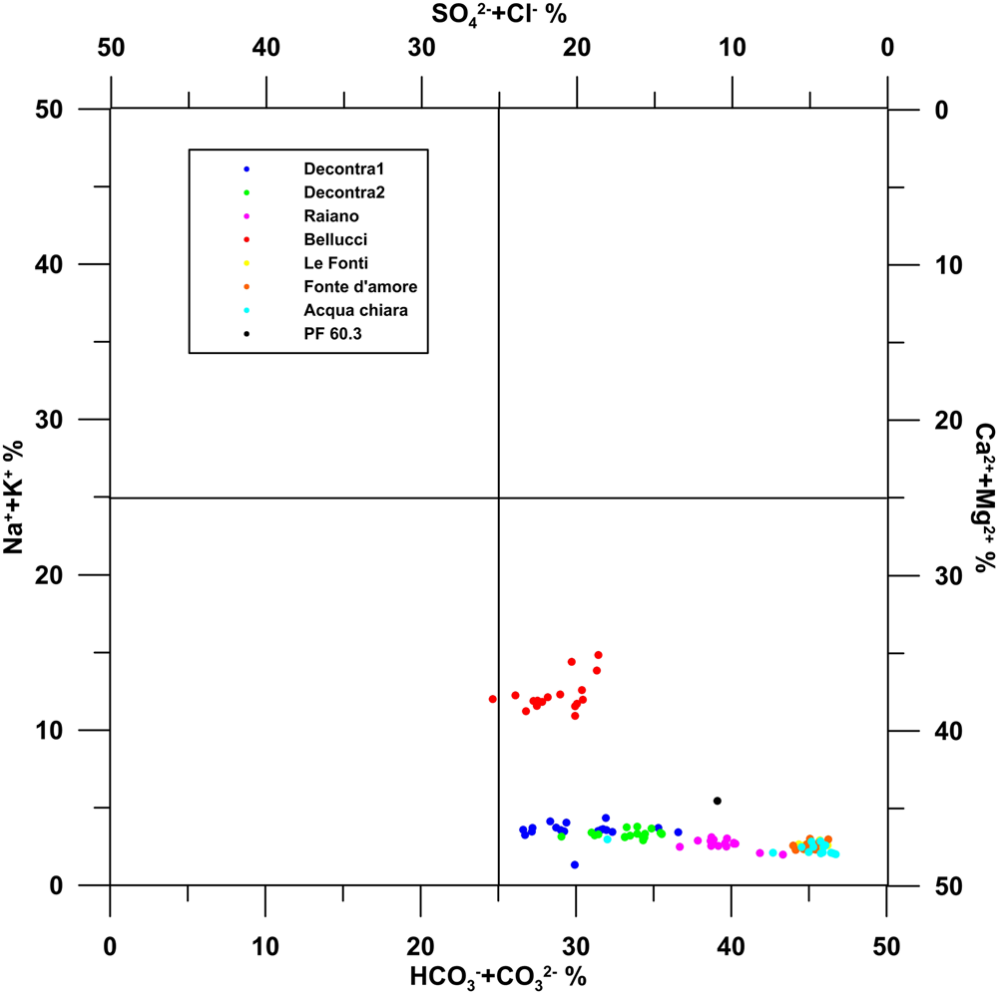
Chebotarev’s classification diagram for groundwater. This figure has been realized using Grapher 7. All groundwater analyzed in this work falls within the calcium-bicarbonate quadrant. See ref.^98^ for the creation method of Chebotarev’s diagram.

δ^18^O and δ^2^H values for the SPTS springs range between −11.1 and −9.4 and between −74.3 and −63.3 respectively (Supplementary Table S3). Each monitored spring shows stable values during the year, confirming that groundwater of the SPTS is characterised by a negligible influence from seasonal recharge water and surface water interaction. Accordingly, the hydrogeochemical features, which are dominated by calcium bicarbonate equilibrium, are usually very steady (Fig. 2). The stability of groundwater flow as inferred from ion content (Fig. 2) allows an optimal evaluation of hydrogeochemical changes due to causes different from hydrogeological ones, i.e. seasonal cycles, surface water/groundwater interaction, and flow or leakage from unsaturated zones.

## Results

Since January 2016, we have monitored - on a monthly basis - some hydrogeochemical parameters in the SPTS (Supplementary Table S3), located about 60 km southeast of the Amatrice-Norcia epicentral area. This hydrogeochemical monitoring followed a few preliminary samplings realised during 2014 and 2015 (Supplementary Table S3). Since the onset of the seismic sequence, we have also monitored the San Chiodo spring in the epicentral area (S8, Fig. 1a). This spring is characterised by high discharge rates and steady regimes typical of regional groundwater flow in the carbonate basal aquifer^74^. We had already sampled this spring once in June 2016 (Supplementary Table S3). Detailed methods and sampling-analytic strategies are fully explained in the Materials and Methods section. The full dataset is reported in Supplementary Table S3.

**Figure 3:**
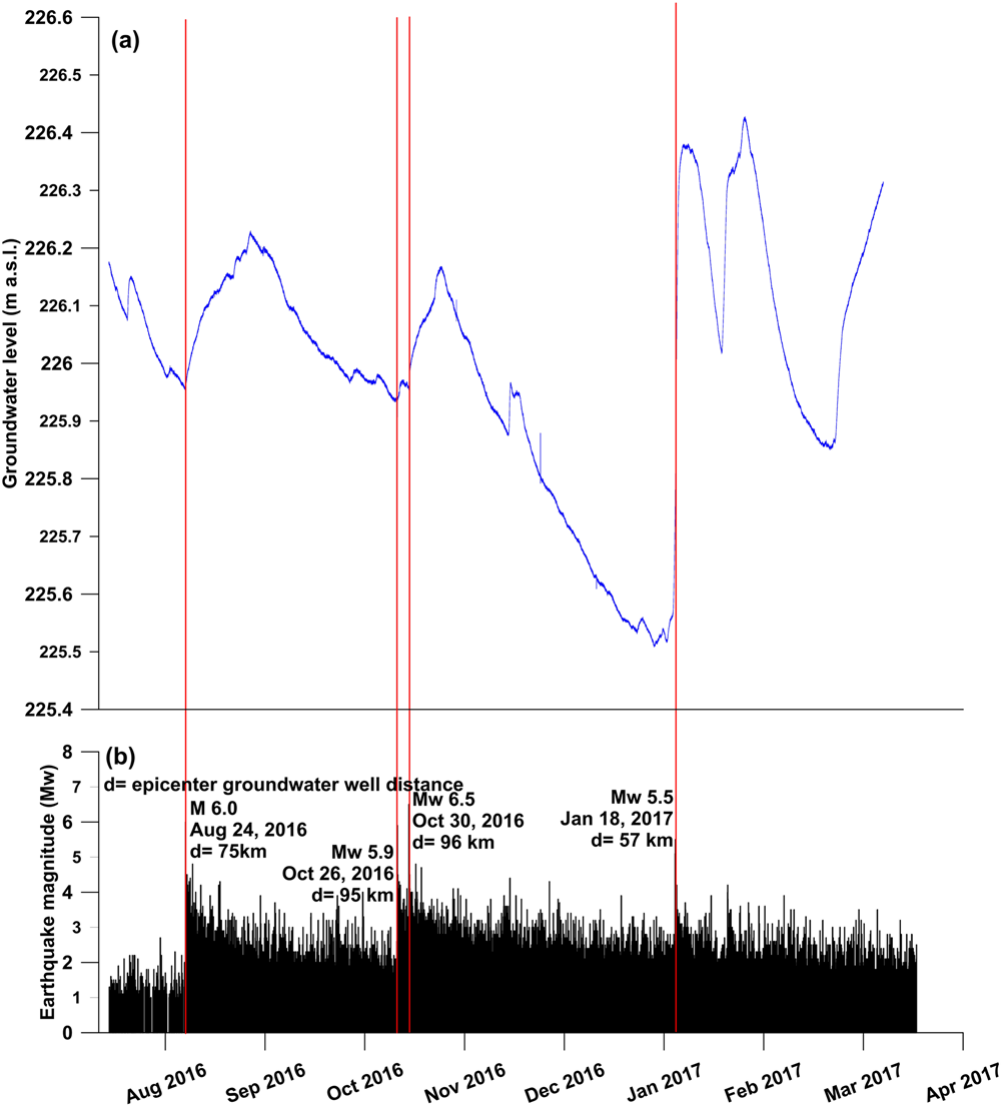
Time series. This figure has been realized using Grapher 7. (**a**) Time series (August 1^st^, 2016-March 31^st^, 2017) of groundwater level recorded in the PF 60.3 well (100 m deep; Fig. 1a). Piezometric data were purposely recorded for this work. Occurrence of main earthquakes (belonging to the 2016–2017 central Apennines sequence; Supplementary Table S1) is shown with vertical red bars. (**b**) Time series (August 1^st^, 2016–March 31^st^, 2017) of magnitude (M_w_) for earthquakes belonging to the 2016–2017 central Apennines sequence. Seismic data are from the INGV database (available online at http://cnt.rm.ingv.it/).

The water table of the monitored aquifer (SPTS) shows a piezometric level in the PF 60.3 well (Fig. 1a; Supplementary Table S2) that is characterised by a normal seasonal depletion phase slightly influenced by smoothed and short recharge events. This trend is suddenly interrupted by three abrupt rises following the four strongest earthquakes (M_w_ 6.0, August 24^th^, 2016; M_w_ 5.9, October 26^th^, 2016; M_w_ 6.5, October 30^th^, 2016; and M_w_ 5.5, January 18^th^, 2017). The October 26^th^ and 30^th^ piezometric rises are partly overlapping each other due to the short time interval between them. The sudden piezometric rises are between a minimum of about 20 cm and a maximum of about 80 cm and occurred within hours to days after the four previously-mentioned earthquakes, which occurred at distances of between 57 and 96 km from the well (Fig. 3). It is worth noting that the highest piezometric rise occurred with the smaller M_w_ 5.5 January 18^th^ seismic event due to its closest distance (about 60 km) from the SPTS.

**Figure 4:**
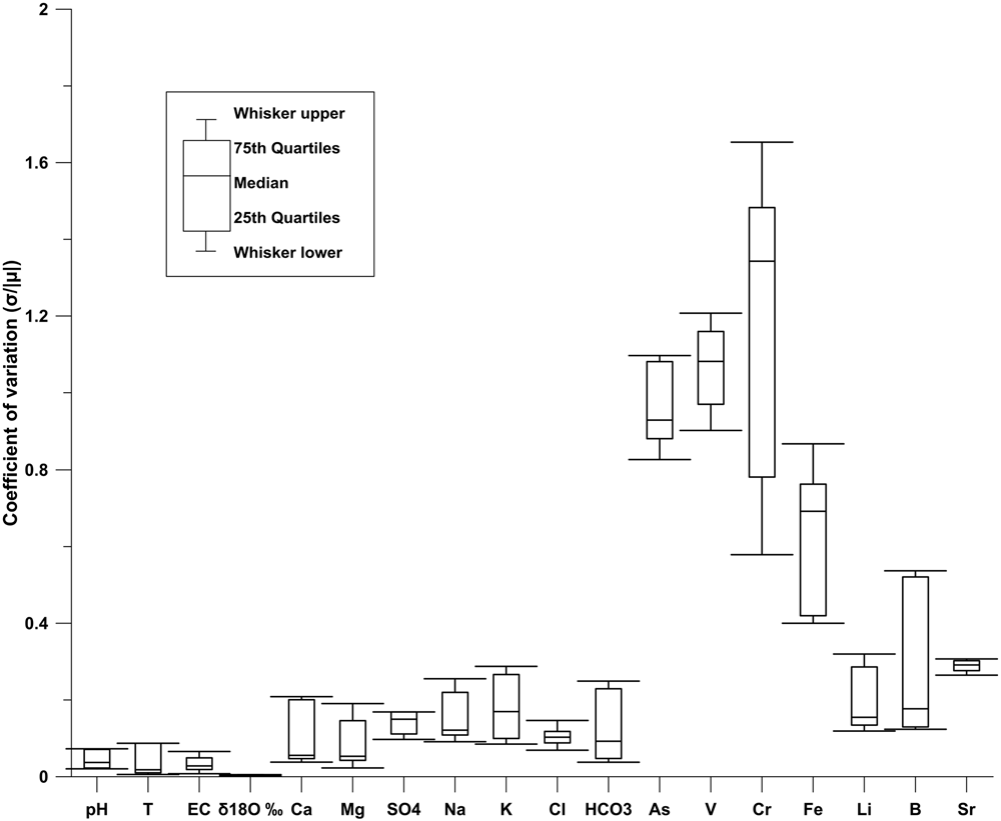
Coefficient of variation. This figure has been realized using Grapher 7. Coefficient of variation for chemical element concentrations and physical-chemical parameters (Supplementary Tables S3) measured in the Sulmona test site springs (Supplementary Table S2) between January 1^st^, 2016 and March 31^st^, 2017. The coefficient of variation corresponds to the ratio between the standard deviation (σ) and the absolute value of the mean (|μ|) of results from the chemical analyses realized on the water samples collected during the considered period (Supplementary Table S3).

From a geochemical point of view, the spring waters monitored in the SPTS are characterised by a substantial invariability with regard to time (between January 2016 and March 2017; Fig. 4) of major elements (Ca, Mg, SO_4_, Na, K, and Cl), stable isotopes (δ^18^O and δ^2^H), and some physical-chemical parameters (temperature and electric conductivity). This invariability is proven by low values of the coefficient of variation (<0.25), which is the ratio between the standard deviation (σ) and the absolute value of the mean (|μ|) of data reported in Supplementary Table S3. A few elements (Li, B, and Sr) are characterised by a slight variability (i.e. coefficient of variation ≤0.4; Fig. 4), whereas four other elements (As, V, Cr, and Fe) are characterised by a marked variability (i.e. coefficient of variation ≥0.4; Fig. 4). For these four elements (As, V, Cr, and Fe), we performed the Shapiro-Wilk normality test^75^ to compare results with the null hypothesis that apparent pre- and post-seismic concentration values are part of a normal distribution. This null hypothesis could be rejected with a p value < 0.05, specifically <10^–16^, 10^−14^, 10^−3^, and 10^−2^ for V, As, Cr, and Fe respectively. The data used to perform the Shapiro-Wilk normality test^75^ are those of Supplementary Table S3.

**Figure 5:**
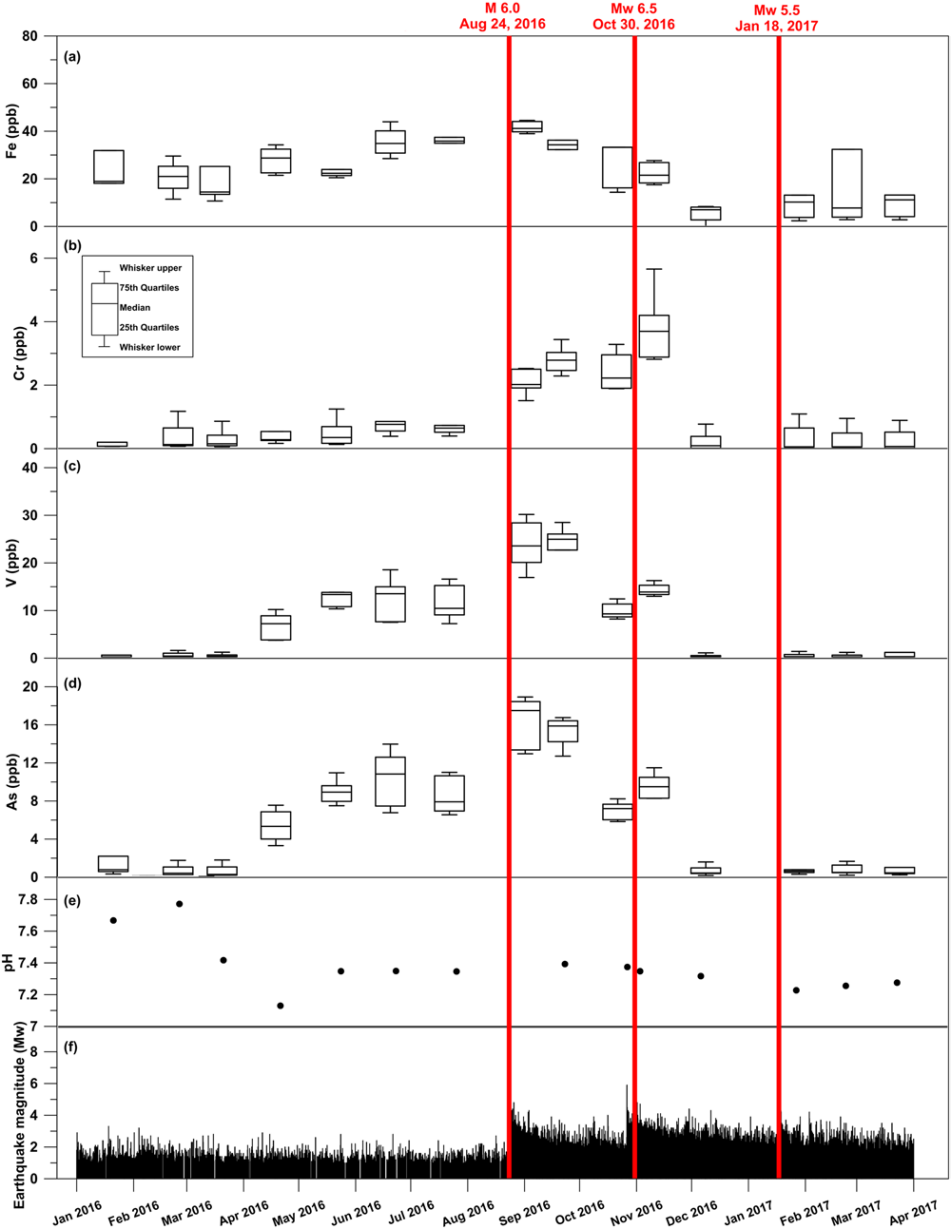
Time series. This figure has been realized using Grapher 7. Time series (January 1^st^, 2016–March 31^st^, 2017) of element concentration, pH (Sulmona test site springs), and earthquake magnitude. Concentration of elements is shown as box-and-whisker plots representing interquartile ranges (25^th^–75^th^ quartiles). Each plot includes geochemical data from the Sulmona test site springs (Supplementary Tables S2 and S3). Seismic data are from the INGV database (available online at http://cnt.rm.ingv.it/). (**a**) Iron, Fe, concentration. (**b**) Chrome, Cr, concentration. (**c**) Vanadium, V, concentration. (**d**) Arsenic, As, concentration. (**e**) Potential of hydrogen, pH. (**f**) Magnitude (M_w_) of earthquakes belonging to the 2016–2017 central Apennines sequence.

The time series of As, V, Cr, and Fe in all springs are plotted in Fig. 5, together with the water pH values only from the four springs affected by deep influx (S1, S2, S3, and S4; Fig. 1a, Supplementary Table S3), and these series are compared with earthquake magnitudes in the same period. From April 2016, prior to the onset of the seismic sequence, As and V were characterised by an evident variability (increase of concentration). A similar temporal pattern can also be observed for Fe, although the increase of concentration is smaller and less marked than for As and V. In contrast, the concentration of Cr increased at the end of August 2016, right after the onset of the seismic sequence. Since December 2016, the concentration of all these four elements started to decrease, returning to the same values as those of March 2016. Past concentrations of As and V, (i.e. water samples collected on 2014 and 2015; Supplementary Table S3) were as low as the concentrations from periods preceding (March 2016) and postdating (December 2016–March 2017) the gain curves (April–November 2016; Fig. 5). In the four selected springs (S1, S2, S3, and S4), pH values were characterised by a peak (~7.9) on February 2016, followed by a minimum on April 2016 (~7.0) and a plateau (~7.4) since May, 2016 (Fig. 5).

**Figure 6:**
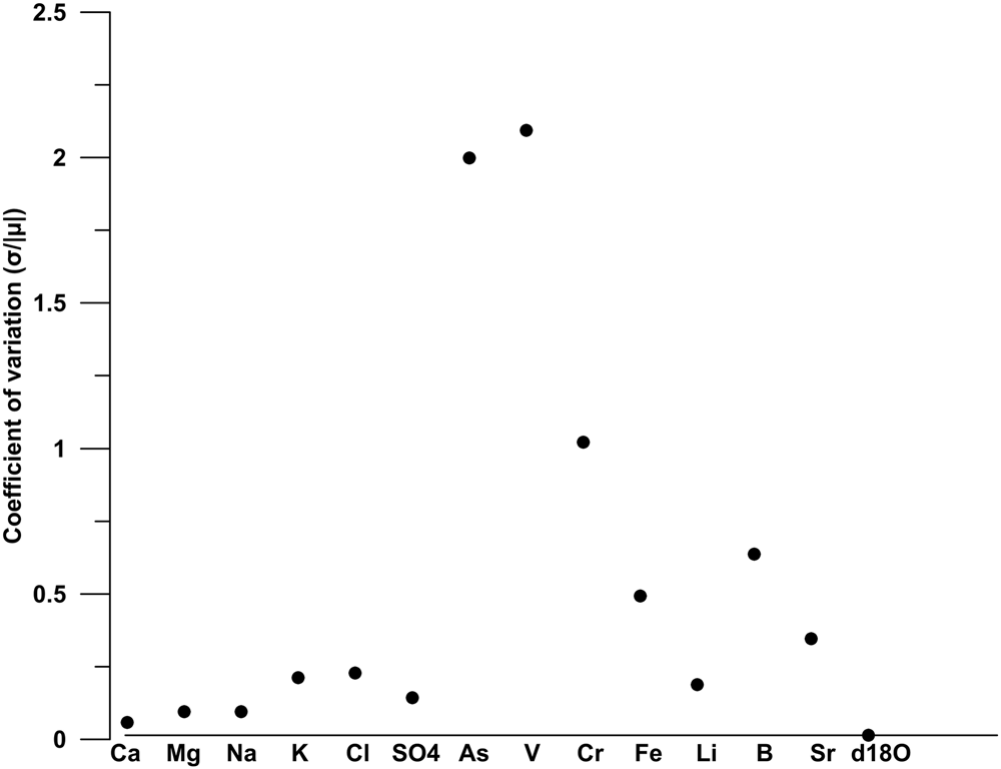
Coefficient of variation. This figure has been realized using Grapher 7. Coefficient of variation for chemical element concentrations and δ^18^O from the San Chiodo spring (see location, S8, in Fig. 1a) between June 1^st^, 2016 and March 31^st^, 2017 (Supplementary Tables S3). The coefficient of variation corresponds to the ratio between the standard deviation (σ) and the absolute value of the mean (|μ|) of results from the chemical analyses realized on the water samples collected during the considered period (Supplementary Table S3).

**Figure 7:**
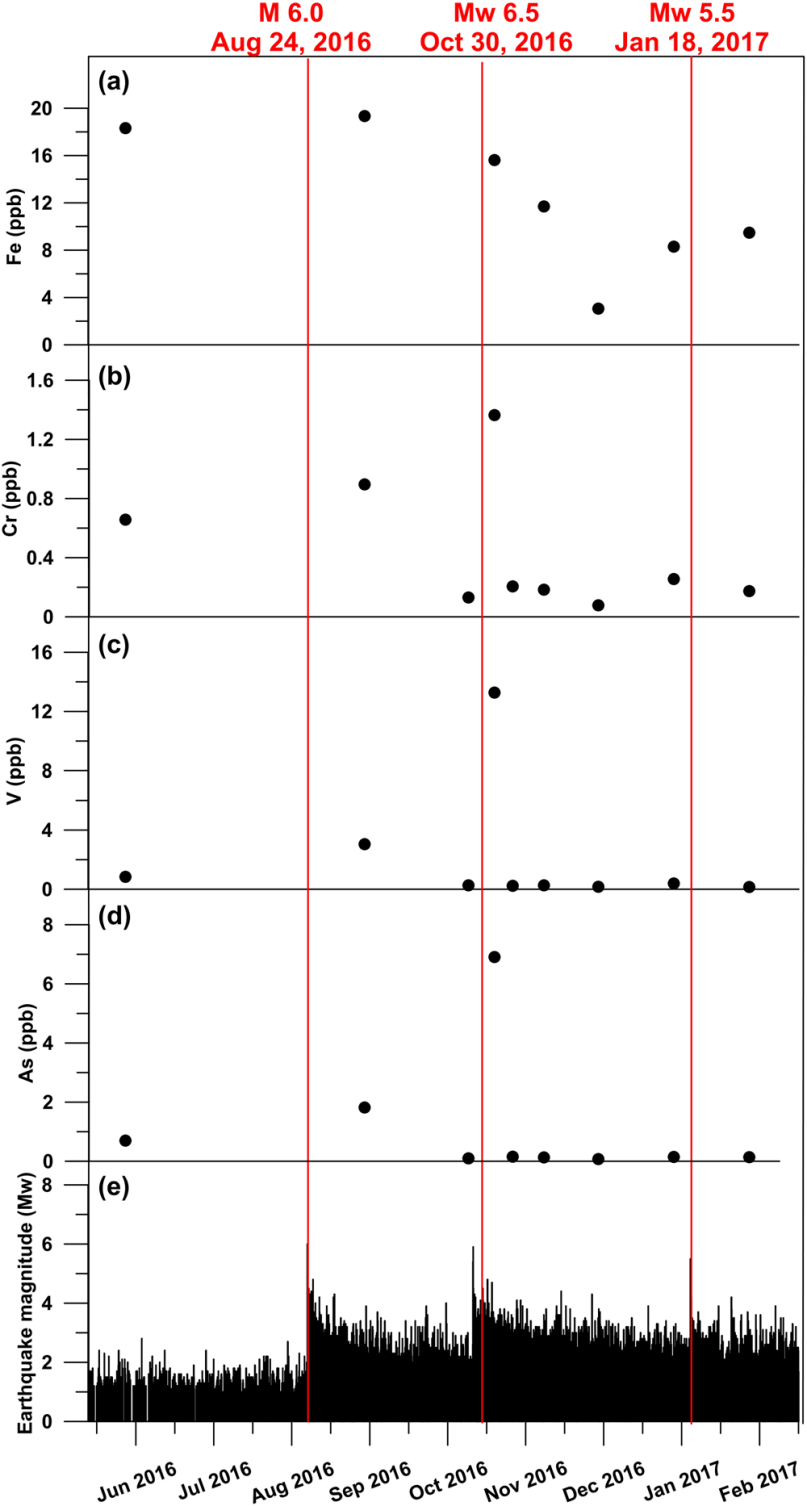
Time series. This figure has been realized using Grapher 7. Time series (June 1^st^, 2016–March 31^st^, 2017) of element concentrations (San Chiodo spring, S8; Supplementary Tables S2 and S3) and earthquake magnitude. Seismic data are from the INGV database (available online at http://cnt.rm.ingv.it/). (**a**) Iron, Fe, concentration. (**b**) Chrome, Cr, concentration. (**c**) Vanadium, V, concentration. (**d**) Arsenic, As, concentration. (**e**) Magnitude (M_w_) of earthquakes belonging to the 2016–2017 central Apennines sequence.

In addition, for the San Chiodo spring, the temporal variability (i.e. after the onset of the 2016–2017 seismic sequence; Supplementary Table S3) of chemical elements and water stable isotopes is very low, except for As, V, and Cr, which show a large coefficient of variation (≥1.0). Fe and B are characterised by a moderate variability (Fig. 6). The San Chiodo spring time series (Fig. 7) of the same elements plotted in Fig. 5, i.e. As, V, Cr, and Fe, does not show clear gain curves, but discrete concentration peaks of As, V, and Cr shortly after the M_w_ 6.5 mainshock of Norcia (October 30^th^, 2016). Starting from the end of November 2016, the As, V, and Cr concentrations significantly reduced down to values lower than those obtained at the onset (September 2016) of the sampling period.

## Discussion

In the central Apennines, responses of the groundwater flow to seismic events have been frequently recorded in historical and recent times^76^. For instance, immediately after the 1980 M_w_ 6.9 Irpinia earthquake, the tapped Capo Sele spring increased its discharge up to four times the average^77^. After the 2009 M_w_ 6.3 L’Aquila earthquake, the Gran Sasso aquifer immediately reacted with changes in discharge, water table levels, and chemical composition^19,78^, which impacted on groundwater biodiversity^79^ as well. Also in the case of the SPTS, the regional aquifer shows clear piezometric responses to the strongest earthquakes of the 2016–2017 sequence (Fig. 3). The piezometric response is clear in terms of post-seismic water table rise, showing, for each considered seismic event, a direct relationship with earthquake magnitude and an inverse relationship with distance from the monitored well (Fig. 3). Moreover, small changes in water table levels have been observed before the seismic events, but their interpretation as precursory events cannot be unambiguously asserted due to several possible causes related to the regular groundwater cycles.

The most significant and novel result from our study is that, in a calcium carbonate aquifer characterised by a steady low content of most metals (Figs 2 and 4), an increase in the content of at least three metals (Cr, Fe, and V) and one metalloid (As) occurred about four months before (As, Fe, and V) or roughly in conjunction with (Cr) the onset of the 2016–2017 seismic sequence in the central Apennines (Fig. 5).

The onset of As, Fe, and V (and subsequently Cr) increment followed a peak of pH and occurred in conjunction with a pH minimum (Fig. 5e), as recorded in the four springs affected by deep fluid contribution. There is no principal source of metals in shallow strata beneath the SPTS (nor beneath the San Chiodo area), which is mainly composed of carbonate rocks and alluvial sediments. Disregarding possible surface sources, and considering that shallow carbonate rocks cannot be the source of the recorded metal-metalloid increment, two main processes can explain our results:

1. Metal and metalloid (in particular As) enrichments can be symptomatic of an influx from a deep endogenic fluid of hydrothermal origin^80,81^. Most metals, in particular trace and transition metals, are generally enriched in hydrothermal fluids, mainly due to complexation at low pH and high temperatures^82^. Trace elements like As can accumulate in the vapor phase due to HS-complexation^83^.
2. As an alternative to a mineralised endogenic fluid, in the study area, possible deep sources for the observed metal ions can be found in bituminous levels containing organic matters^84^ and/or in phyllites and quartzites located at a depth of about 10 km^40^ (Fig. 1), corresponding to the earthquake focal depth. In this case, the enrichment in metal and metalloid elements would result from an interaction between rocks and fluids, the latter made aggressive (i.e. see the pre-seismic pH decrease in Fig. 5e) through, for instance, the influx of deep CO_2_ trapped beneath the evaporites.

The presence of endogenic CO_2_ and metal-rich hydrothermal fluids beneath the Apennines is well-known and largely testified in groundwater showing deep contributions^44,45,66^. In the investigated setting, the presence of anhydritic evaporites at the base of Meso-Cenozoic carbonate deposits commonly constitutes an impermeable barrier to endogenic fluids, including CO_2_ and hydrocarbons^43,46,47,84,85^. A recent geophysical study, in particular, showed a marked increase of V_p_/V_s_ below the SPTS, at a depth of 10–15 km, likely in relation to endogenic fluid trapped below the evaporitic sealing level^84^. Therefore, we believe that, in the study area, this deep barrier may have been disrupted by pre-seismic dilatational processes (e.g. refs^5,29,86^) affecting a large area in the Apennine chain, including the region along and across the Mt. Morrone Fault (Fig. 1c), and this process eventually enhanced the influx and ascent of CO_2_ and/or other metal-rich endogenic fluids.

In the study region, pre- and co-seismic CO_2_ increases have been previously documented^87,88^. Accordingly, we interpret the observed content increases for As, V, and, subordinately, Fe in the monitored springs as potential seismic precursors, at least in the study area. Deep CO_2_ influx should indeed contribute to temporarily lower pH (as observed in Fig. 5) in the regional carbonate aquifer located above the evaporitic levels, with a very clear influence in springs having direct deep contributions (S1, S2, S3, and S4). The following buffering of pH (as observed in Fig. 5) would enhance solubility and mobility of some chemical elements, particularly As, V, Cr, and Fe, and perhaps other metals (e.g. Sr; Supplementary Table S3). There are several reasons why the pH might increase, but the most important in this context is the uptake of protons by mineral weathering and ion-exchange reactions, combined with the inputs of high-pH geothermal water. At near-neutral pH, the solubility of most trace-metal cations is severely limited (e.g. adsorption to hydrous metal oxides, clay, or organic matter). In contrast, most oxyanions, including arsenate, vanadate, and chromate, tend to become less strongly sorbed as the pH increases^89^. Therefore, the oxyanion forming elements such as Cr, As, and V are some of the most common trace contaminants in groundwater. Chromium can similarly be mobilised as stable Cr(VI) oxyanion species under oxidising conditions, but forms cationic Cr(III) species in reducing environments, and hence behaves like other trace cations (i.e. it is relatively immobile at near-neutral pH values). In this context, the most important possible geochemical triggers involved in the release of As, V, and Cr appear to be the desorption/dissolution of these trace elements from oxide minerals, particularly iron oxides. This notion is consistent with the observed simultaneous increase of As, V, and Fe (Fig. 5). These are, in part, related to physical factors, such as the rate of diffusion of gases through the sediment and the rate of sedimentation, in part due to the extent of organic matter. Moreover, bituminous-asphaltic residues often fill the intensely fractured Triassic-Miocene carbonatic and calcareous-marly rocks^84^ related to paleogeothermal evolution of the two sedimentary-structural domains developed in the western sector of the central Appenines^90^.

Our data (Supplementary Table S3) shows that Fe and Cr contents were rather high on November 2014, and that for Fe was also rather high on March 2015. These contents, in particular, are similar to the pre-seismic ones for Fe (around 35–40 ppb) and the co- and post-seismic ones for Cr (>2 ppb). These results suggest that, in our study, As and V should be considered as the most reliable seismic precursors. The increase in Cr content, in particular, is merely co- or post-seismic, at least concerning the 2016–2017 sequence (Fig. 5), whereas the content of Fe in groundwater is known to be sensitive to various environmental processes^91^, including the seismic cycle (Fig. 5; e.g. ref.^26^). Obviously, also the monthly frequency of our sampling played a role in determining the temporal precursors of earthquakes.

From a geochemical point of view, it is noteworthy that, in previous normal faulting cases, such as those from Iceland, the potential seismic precursors identified in groundwater were also metals and metalloids, including Cu, Zn, Mn, Cr, and Fe^26^, and Si, Na, and Ca^31^. In addition, for the normal faulting case of the San Chiodo spring, the elements potentially sensible to seismic cycles were metals and metalloids such as V, Cr, and As (Figs 6 and 7). Although the chemical analogy between these cases (i.e. Iceland and San Chiodo) and the SPTS suggests that metal-metalloid concentrations in groundwater is a promising field of research for the science of seismic precursors, the type of metals-metalloids involved, as well as the variability of the related temporal series (see Fig. 5 vs. Fig. 7), show that such seismic precursors, if real, are surely site-specific (i.e. geology- and hydrogeology dependent). Accordingly, to obtain significant results, the selection of the monitoring springs must be based on a robust conceptual model of groundwater flow coupled with a detailed geological-structural setting, as is the case of the study area, where preliminary sampling and surveys started two years before the systematic monitoring activity.

It is also noteworthy that the spatial (tens of km from the epicentre) and temporal (months) scales of hydrogeochemical processes and changes recorded in this paper (Fig. 5), as well as the earthquake magnitude ranges (5.5 ≤ M_w_ ≤ 6.5) and tectonic setting (extensional), are similar to those involved in similar processes previously analysed elsewhere^24–26,31,92–94^. In addition, the conceptual model of contribution to groundwater regional flow by deep raising fluids in pre-seismic periods is in agreement with the seismic model of graviquakes^86^. In fact, the initiation of gradual collapse of the brittle upper crustal prism can be inferred as due to the closure of a multitude of microfractures developed during the interseismic period above the brittle-ductile transition. The closure of the fractures set must have squeezed out the fluids content in the secondary porosity, both during and after the mainshock, but possibly also before it.

We conclude that:

1. Hydrogeochemical monitoring of seven springs and one well in central Apennines during 2016 and 2017 allowed the discovery of two potential seismic precursors, which are the changes in As and V content in deeply-sourced groundwater.
2. Changes in the content of other metals and metalloids, such as Cr and Fe, are potentially sensitive to the seismic cycles, but their precursory role has to be further investigated and understood.
3. Previous studies^26,31^ documented similar and consistent observations in Iceland prior than intermediate-magnitude earthquakes; however, the precursory chemical elements were different from ours. These partly different results show the site-specificity of geochemical precursors and therefore the need for this type of study at the local-to-regional scale over the long term.
4. In addition to the identification of some seismic precursors, the novelty of our study is in finding a link between geochemical precursors, crustal tectonic setting, and deep pre-seismic geological processes, including dilation, influx of hydrothermal fluids and/or deeply-trapped CO_2_, fluid-rock interaction, and fluid ascent.

## Methods and Materials

We monitored and sampled seven springs, a 100 m deep well in the Sulmona Plain Test Site (SPTS), and one spring in the epicentral area of the 2016–2017 seismic sequence of central Apennines (Fig. 1a). All data is reported in Figs 2–7 and Supplementary Tables S2 and S3.

Hydrogeological-hydrogeochemical analyses included the following determinations: (1) well piezometry, (2) physics and chemistry of spring waters, and (3) stable isotope chemistry of spring waters.

1. The piezometric level in the PF 60.3 well was monitored using the OTT ecoLog 800 probe (resolution 1 mm), placed into the well at a depth of 25 m. Acquisition frequency was every 5 minutes, and data remote transmission occurred every 8 hours via GSM.
2. Water temperature (resolution 0.1 °C), electrical conductivity (EC), and pH (resolution 0.001) were measured in the field using the WTW Multi 3420 probe. The chemical composition was determined using standard analytical methods^95^. Following filtration in the field (0.45 μm), major ions were analysed with a Chromeleon Dionex (precision ±2%). An ICS 1100 was used for analysing cations, and a Dionex ICS5000 was used for analysing anions. Concentrations of minor and trace elements were measured using an ICP-MS (X Series 2 Thermo Fisher Scientific) following filtration (0.45 μm) and acidification with 0.1 N HCl in the field. The analyses were performed at the Geochemical Laboratory, Department of Earth Sciences, Sapienza University of Rome (Italy). The analytical accuracy of these methods ranged from 2% to 5%. An internal standard, Rh, was used to correct the ICP-MS instrumental drift. Ultrapure water (Millipore, Milli-Q, 16 MΩ cm) was used in the preparation of blanks, standard solutions, and sample dilutions.
3. δ^18^O and δ^2^H of water molecules were determined at the Isotope Geochemical Laboratory of Parma University, using the Finnigan Delta Plusmass spectrometer. The results are reported in δ‰ units vs. the international V-SMOW standard^96^. The standard deviations of the measurements were equal to approximately ±1‰ for δ^2^H and to ±0.2‰ for δ^18^O. Results are reported in Supplementary Table S3.

The Shapiro-Wilk normality test^75^ was performed using the SPTS data from Supplementary Table S3. Data was processed with the Pro-ULC software (https://www.epa.gov/land-research/proucl-software). This test was used to compare with the null hypothesis that apparent pre- and post-seismic values were part of a normal distribution. This null hypothesis could be rejected with a p value < 0.05 for V, As, Cr, and Fe. In this sense, p values were smaller than 10^−16^, 10^−14^, 10^−3^, and 10^−2^ for V, As, Cr, and Fe respectively.

## Electronic supplementary material

Dataset 1
