# Supplementary material for: Hydrogeochemical changes before and during the 2016 Amatrice-Norcia seismic sequence (central Italy)
Source: Sci Rep. 2017 Sep 15;7:11735. doi: 10.1038/s41598-017-11990-8 (PMC5601465; doi:10.1038/s41598-017-11990-8)
Supplement: Supplementary file 1 — Dataset 1 [file 41598_2017_11990_MOESM1_ESM.doc]

**Hydrogeochemical changes before and during the 2016 Amatrice-Norcia seismic sequence (central Italy).**

Marino Domenico Barberio^(1)^, Maurizio Barbieri^(1)^, Andrea Billi^(2,*)^, Carlo Doglioni^(1,3)^, and Marco Petitta^(1)^

^(1)^ Dipartimento di Scienze della Terra, Sapienza University of Rome, Italy

^(2)^ Consiglio Nazionale delle Ricerche, IGAG, Rome, Italy

^(3)^ Istituto Nazionale di Geofisica e Vulcanologia, Rome, Italy

^(*)^ Corresponding author: Andrea Billi, Consiglio Nazionale delle Ricerche, IGAG, c.o. Dipartimento di Scienze della Terra, Sapienza University of Rome, P.le A. Moro 5, 00185, Rome, Italy. Phone: +39 06-49914955. Email: [andrea.billi@cnr.it](mailto:andrea.billi@cnr.it)

**Table S1.** Location and data concerning M_w_ ≥ 5.0 earthquakes belonging to the 2016-2017 seismic sequence of central Apennines.

| **Date/Time (UTC)** | **Lat.(°)** | **Long.(°)** | **Depth (km)** | **Magnitude (Mw)** |
| --- | --- | --- | --- | --- |
| **1/18/17 1:33 PM** | 42.477 | 13.281 | 10 | 5 |
| **1/18/17 10:25 AM** | 42.494 | 13.311 | 8.9 | 5.4 |
| **1/18/17 10:14 AM** | 42.529 | 13.282 | 9.1 | 5.5 |
| **1/18/17 9:25 AM** | 42.547 | 13.262 | 9.2 | 5.1 |
| **10/30/16 6:40 AM** | 42.832 | 13.111 | 9.2 | 6.5 |
| **10/26/16 7:18 PM** | 42.909 | 13.129 | 7.5 | 5.9 |
| **10/26/16 5:10 PM** | 42.88 | 13.128 | 8.7 | 5.4 |
| **8/24/16 2:33 AM** | 42.792 | 13.151 | 8 | 5.4 |
| **8/24/16 1:36 AM** | 42.698 | 13.234 | 8.1 | 6 |

**Table S2.** Location and data concerning the well and springs monitored and analyzed in this work.

| **Type** | **ID** | **Name** | **Lat. (°)** | **Long. (°)** | **Elevation (meters a.s.l.)** | **Chemical Character** |
| --- | --- | --- | --- | --- | --- | --- |
| **Spring** | S1 | Decontra1 | 42.178 | 13.837 | 245 | Sulphurous |
| **Spring** | S2 | Decontra 2 | 42.184 | 13.839 | 243 | Sulphurous |
| **Spring** | S3 | Bellucci | 42.192 | 13.846 | 235 | Sulphurous |
| **Spring** | S4 | Raiano | 42.108 | 13.808 | 275 | Sulphurous |
| **Spring** | S5 | Acqua Chiara | 42.072 | 13.893 | 319 | Calcium Bicarbonate |
| **Spring** | S6 | Le Fonti | 42.118 | 13.884 | 315 | Calcium Bicarbonate |
| **Spring** | S7 | Fonte d'Amore | 42.083 | 13.929 | 359 | Calcium Bicarbonate |
| **Spring** | S8 | San Chiodo | 42.893 | 13.161 | 760 | Calcium Bicarbonate |
| **Well** | PF 60.3 | Bussi Officine | 42.197 | 13.852 | 238 | Calcium Bicarbonate |

**Table S3.** Chemical element concentrations and physical-chemical parameters measured in the considered springs.

| S 1 |  |  |  |  |  |  |  |  |  |  |  |  |  |  |  |  |  |  |  |
| --- | --- | --- | --- | --- | --- | --- | --- | --- | --- | --- | --- | --- | --- | --- | --- | --- | --- | --- | --- |
| Date | pH | T (°C) | EC (µS/cm) | δ^18^O | δD | Ca (ppm) | Mg (ppm) | Na (ppm) | K(ppm) | Cl (ppm) | SO_4_ (ppm) | HCO_3_ | As (ppb) | V (ppb) | Cr (ppb) | Fe (ppb) | Li (ppb) | B (ppb) | Sr (ppb) |
| November 5, 2014 | 7.23 | 13.8 | 887 | -10.1 | -67.7 | 143.3 | 39.5 | 15.1 | 4.1 | 21.9 | 114.2 | 498 | 0.57 | 0.24 | 1.80 | 43.45 | 2.17 | 40.31 | 1856.00 |
| March 12, 2015 | - | 13.8 | 762 | -10.0 | -66.8 | 120.2 | 35.2 | 3.2 | 3.8 | 24.5 | 143.0 | 334 | 0.91 | 0.09 | 0.00 | 36.76 | 2.78 | 38.68 | 779.20 |
| December 17, 2015 | 7.36 | 13.7 | 770 | -10.2 | -68.4 | 112.7 | 31.3 | 12.3 | 2.6 | 17.3 | 125.9 | 347 | 0.57 | 0.15 | 0.03 | 26.44 | 19.76 | 184.50 | 741.90 |
| January 21, 2016 | 7.66 | 13.6 | 783 | -10.3 | -68.8 | 112.3 | 32.1 | 12.9 | 3.7 | 20.7 | 97.6 | 384 | 0.98 | 0.21 | 0.08 | 18.12 | 15.86 | 167.10 | 662.80 |
| February 26, 2016 | 7.53 | 13.6 | 799 | -10.2 | -69.2 | 114.1 | 33.0 | 14.9 | 4.2 | 21.1 | 162.1 | 317 | 1.07 | 0.19 | 0.10 | 16.01 | 15.63 | 163.60 | 647.60 |
| March 21, 2016 | 7.36 | 13.7 | 809 | -10.1 | -68.3 | 125.1 | 34.4 | 17.0 | 4.7 | 19.1 | 146.7 | 387 | 1.08 | 0.21 | 0.06 | 14.46 | 13.19 | 141.10 | 560.00 |
| April 21, 2016 | 7.35 | 13.7 | 819 | -10.2 | -68.1 | 117.8 | 32.0 | 15.3 | 3.2 | 22.1 | 153.5 | 332 | 4.01 | 3.76 | 0.28 | 28.73 | 17.16 | 179.00 | 1059.00 |
| May 24, 2016 | 7.50 | 13.6 | 820 | -10.2 | -67.3 | 122.3 | 33.2 | 12.7 | 4.2 | 19.5 | 142.9 | 364 | 8.41 | 10.36 | 0.13 | 22.33 | 15.94 | 160.80 | 944.20 |
| June 23, 2016 | 7.27 | 13.9 | 823 | -10.2 | -68.2 | 121.4 | 34.6 | 13.8 | 4.4 | 24.0 | 164.1 | 337 | 7.47 | 7.65 | 0.60 | 43.96 | 16.33 | 191.30 | 1099.00 |
| July 26, 2016 | 7.24 | 14 | 825 | -10.1 | -67.3 | 122.3 | 34.7 | 13.3 | 4.3 | 24.9 | 183.2 | 313 | 7.87 | 9.49 | 0.56 | 37.44 | 16.72 | 196.40 | 1099.00 |
| August 29, 2016 | - | 13.9 | 819 | -10.2 | -67.3 | 111.6 | 31.7 | 11.7 | 3.7 | 20.1 | 165.4 | 291 | 13.36 | 16.94 | 2.00 | 44.08 | 16.83 | 201.40 | 1128.00 |
| September 23, 2016 | 7.30 | 13.8 | 827 | -10.2 | -67.7 | 113.1 | 32.3 | 12.1 | 3.7 | 21.9 | 148.3 | 319 | 12.71 | 16.40 | 2.65 | 36.24 | 16.95 | 197.60 | 1130.00 |
| October 27, 2016 | 7.24 | 13.7 | 841 | -10.2 | -67.9 | 123.5 | 34.9 | 13.6 | 3.9 | 18.9 | 170.4 | 345 | 7.20 | 9.30 | 2.96 | 33.21 | 17.11 | 178.90 | 1015.00 |
| November 3, 2016 | 7.33 | 13.7 | 828 | -10.2 | -67.6 | 128.4 | 34.6 | 14.0 | 4.0 | 24.0 | 143.3 | 385 | 9.90 | 13.37 | 4.20 | 21.51 | 18.93 | 181.00 | 1132.00 |
| December 6, 2016 | 7.29 | 13.6 | 832 | -10.2 | -68.2 | 115.5 | 33.4 | 13.4 | 3.8 | 22.5 | 170.9 | 305 | 0.98 | 0.13 | 0.01 | 7.01 | 17.31 | 200.30 | 507.80 |
| January 27, 2017 | 7.16 | 13.5 | 841 | - | - | 112.4 | 33.0 | 11.2 | 3.4 | 20.7 | 170.9 | 290 | 0.79 | 0.22 | 0.04 | 10.24 | 18.98 | 228.30 | 465.80 |
| February 23, 2017 | 7.16 | 13.7 | 836 | - | - | 132.4 | 36.9 | 14.7 | 3.9 | 19.4 | 153.5 | 405 | 1.27 | 0.14 | 0.04 | 32.38 | 14.89 | 195.10 | - |
| March 23, 2017 | 7.141 | 13.6 | 843 | - | - | 127.5 | 34.8 | 13.9 | 4.3 | 22.1 | 144.4 | 385 | 1.03 | 0.10 | 0.02 | 11.17 | 11.56 | 152.90 | - |
|  |  |  |  |  |  |  |  |  |  |  |  |  |  |  |  |  |  |  |  |
| S 2 |  |  |  |  |  |  |  |  |  |  |  |  |  |  |  |  |  |  |  |
| Date | pH | T (°C) | EC (µS/cm) | δ^18^O | δD | Ca (ppm) | Mg (ppm) | Na (ppm) | K(ppm) | Cl (ppm) | SO_4_ (ppm) | HCO_3_ | As (ppb) | V (ppb) | Cr (ppb) | Fe (ppb) | Li (ppb) | B (ppb) | Sr (ppb) |
| November 5, 2014 | 7.42 | 12.9 | 690 | -10.2 | -68.8 | 116.0 | 27.1 | 10.5 | 2.6 | 16.2 | 106.1 | 359 | 0.34 | 0.25 | 0.31 | 88.37 | 0.72 | 23.93 | 1173.00 |
| March 12, 2015 | - | 12.4 | 697 | -10.3 | -68.1 | 99.0 | 25.5 | 9.4 | 2.9 | 17.9 | 111.3 | 286 | 0.30 | 0.09 | 0.00 | 77.87 | 1.10 | 34.57 | 522.40 |
| December 17, 2015 | 7.59 | 11.7 | 597 | -10.4 | -69.1 | 84.2 | 20.0 | 8.1 | 2.3 | 12.7 | 69.8 | 272 | 0.06 | 0.13 | 0.05 | 37.95 | 10.59 | 104.00 | 455.30 |
| January 21, 2016 | 7.89 | 10.8 | 617 | -10.4 | -69.2 | 93.2 | 22.3 | 9.0 | 2.6 | 15.5 | 75.6 | 301 | 0.34 | 0.23 | 0.08 | 31.89 | 9.03 | 96.42 | 421.90 |
| February 26, 2016 | 8.04 | 11.8 | 627 | -10.4 | -69.9 | 93.9 | 23.2 | 10.3 | 2.6 | 14.8 | 83.3 | 303 | 0.24 | 0.22 | 0.07 | 29.56 | 9.11 | 97.86 | 409.90 |
| March 21, 2016 | 7.58 | 11.9 | 639 | -10.2 | -68.2 | 101.1 | 23.4 | 11.6 | 2.5 | 16.4 | 94.1 | 312 | 0.24 | 0.24 | 0.11 | 56.12 | 7.73 | 81.40 | 352.40 |
| April 21, 2016 | 7.00 | 12.3 | 632 | -10.4 | -68.2 | 98.1 | 21.8 | 10.1 | 1.7 | 14.4 | 80.7 | 310 | 5.34 | 6.95 | 0.17 | 34.26 | 9.91 | 103.50 | 656.20 |
| May 24, 2016 | 7.68 | 12.6 | 637 | -10.4 | -67.7 | 96.8 | 22.3 | 7.9 | 2.5 | 15.0 | 86.1 | 297 | 7.51 | 10.81 | 0.30 | 32.86 | 9.47 | 93.99 | 606.90 |
| June 23, 2016 | 7.45 | 13 | 638 | -10.3 | -68.4 | 95.5 | 23.0 | 8.7 | 2.8 | 15.3 | 92.0 | 291 | 6.77 | 7.54 | 0.39 | 28.51 | 9.61 | 111.60 | 702.00 |
| July 26, 2016 | 7.43 | 13.3 | 644 | -10.4 | -68.2 | 98.4 | 24.0 | 8.8 | 2.7 | 16.7 | 96.2 | 297 | 8.63 | 11.55 | 0.52 | 43.92 | 9.79 | 113.60 | 699.20 |
| August 29, 2016 | - | 12.6 | 640 | -10.3 | -67.5 | 86.4 | 20.3 | 7.7 | 2.3 | 13.8 | 84.6 | 258 | 18.44 | 28.41 | 1.91 | 39.75 | 9.48 | 111.50 | 705.20 |
| September 23, 2016 | 7.41 | 12.5 | 662 | -10.3 | -68.4 | 96.3 | 23.8 | 8.7 | 2.5 | 13.7 | 90.3 | 302 | 16.42 | 25.15 | 3.03 | 34.28 | 10.39 | 127.80 | 801.60 |
| October 27, 2016 | 7.58 | 13.6 | 642 | -9.4 | -63.3 | 95.2 | 22.0 | 9.4 | 4.7 | 16.3 | 91.9 | 288 | 6.03 | 8.67 | 1.90 | 61.40 | 9.26 | 108.80 | 591.30 |
| November 3, 2016 | 7.38 | 12.5 | 700 | -10.3 | -68.7 | 112.1 | 27.0 | 10.8 | 3.4 | 18.9 | 103.6 | 347 | 11.49 | 16.29 | 3.70 | 27.67 | 12.57 | 124.20 | 832.10 |
| December 6, 2016 | 7.23 | 9.5 | 710 | -10.3 | -68.7 | 101.3 | 25.4 | 10.3 | 2.8 | 17.9 | 115.6 | 290 | 0.47 | 0.08 | 0.02 | 8.37 | 12.06 | 144.40 | 380.00 |
| January 27, 2017 | 7.18 | 11.3 | 695 | - | - | 97.7 | 25.6 | 9.0 | 2.7 | 18.9 | 124.2 | 264 | 0.64 | 0.13 | 0.04 | 13.14 | 12.46 | 156.50 | 324.40 |
| February 23, 2017 | 7.29 | 12.2 | 701 | - | - | 113.1 | 27.9 | 11.0 | 3.2 | 15.2 | 106.5 | 358 | 0.47 | 0.13 | 0.05 | 57.30 | 8.74 | 121.70 | - |
| March 23, 2017 | 7.382 | 13.3 | 699 | - | - | 107.2 | 25.8 | 10.3 | 2.9 | 17.0 | 119.4 | 307 | 0.38 | 0.10 | 0.00 | 52.77 | 7.56 | 110.80 | - |
|  |  |  |  |  |  |  |  |  |  |  |  |  |  |  |  |  |  |  |  |
| S 3 |  |  |  |  |  |  |  |  |  |  |  |  |  |  |  |  |  |  |  |
| Date | pH | T (°C) | EC (µS/cm) | δ^18^O | δD | Ca (ppm) | Mg (ppm) | Na (ppm) | K(ppm) | Cl (ppm) | SO_4_ (ppm) | HCO_3_ | As (ppb) | V (ppb) | Cr (ppb) | Fe (ppb) | Li (ppb) | B (ppb) | Sr (ppb) |
| November 5, 2014 | 7.56 | 13 | 808 | -10.0 | -66.6 | 104.5 | 21.1 | 49.1 | 2.6 | 75.3 | 72.5 | 248 | 0.50 | 0.41 | 6.52 | 95.42 | 0.42 | 17.50 | 1137.00 |
| March 12, 2015 | - | 12.9 | 754 | -10.0 | -66.3 | 92.5 | 19.9 | 43.9 | 2.6 | 87.7 | 81.9 | 396 | 0.38 | 0.13 | 0.00 | 87.67 | 0.46 | 16.46 | 539.60 |
| December 17, 2015 | 7.59 | 12.9 | 762 | -10.0 | -66.3 | 86.8 | 18.9 | 41.7 | 2.6 | 69.5 | 68.8 | 267 | 0.31 | 0.21 | 0.05 | 21.01 | 11.92 | 119.40 | 617.30 |
| January 21, 2016 | 7.76 | 12.9 | 765 | -10.0 | -67.2 | 93.4 | 20.4 | 45.8 | 2.9 | 74.3 | 69.0 | 297 | 0.60 | 0.26 | 0.07 | 18.93 | 8.77 | 97.55 | 497.50 |
| February 26, 2016 | 7.95 | 12.9 | 768 | -10.0 | -67.3 | 98.4 | 20.2 | 56.0 | 3.2 | 71.2 | 66.4 | 348 | 0.42 | 0.32 | 0.10 | 25.27 | 8.57 | 96.84 | 480.30 |
| March 21, 2016 | 7.57 | 12.9 | 771 | -10.0 | -66.7 | 97.8 | 20.6 | 59.3 | 3.1 | 75.0 | 78.3 | 335 | 0.41 | 0.30 | 0.09 | 25.19 | 6.98 | 74.22 | 391.60 |
| April 21, 2016 | 7.29 | 13 | 767 | -10.0 | -66.2 | 98.1 | 19.0 | 61.2 | 2.7 | 70.7 | 68.2 | 353 | 3.32 | 3.84 | 0.26 | 32.45 | 10.13 | 106.40 | 842.90 |
| May 24, 2016 | 7.21 | 13 | 718 | -10.0 | -66.2 | 94.9 | 20.0 | 44.1 | 2.8 | 75.5 | 78.5 | 281 | 7.96 | 11.02 | 0.17 | 23.93 | 8.86 | 86.15 | 682.60 |
| June 23, 2016 | 7.45 | 13.1 | 770 | -10.0 | -66.2 | 88.3 | 20.5 | 45.3 | 3.0 | 67.4 | 62.2 | 302 | 11.32 | 13.54 | 0.56 | 30.81 | 8.68 | 100.50 | 781.30 |
| July 26, 2016 | 7.45 | 13.1 | 770 | -10.0 | -66.1 | 96.3 | 21.0 | 46.7 | 3.4 | 89.2 | 77.8 | 276 | 7.92 | 10.46 | 0.40 | 34.95 | 9.16 | 106.50 | 810.80 |
| August 29, 2016 | - | 13 | 771 | -10.0 | -66.4 | 90.7 | 19.7 | 42.3 | 2.9 | 71.3 | 79.6 | 268 | 18.92 | 30.18 | 2.08 | 41.14 | 9.44 | 112.30 | 834.20 |
| September 23, 2016 | 7.47 | 12.9 | 772 | -10.0 | -66.4 | 90.1 | 19.9 | 40.6 | 2.8 | 63.7 | 67.3 | 291 | 15.91 | 26.07 | 2.92 | 44.16 | 10.00 | 117.60 | 903.50 |
| October 27, 2016 | 7.47 | 12.9 | 780 | -10.0 | -66.1 | 97.9 | 21.3 | 45.9 | 3.4 | 63.5 | 77.8 | 324 | 8.23 | 12.45 | 2.22 | 33.27 | 9.50 | 92.53 | 730.40 |
| November 3, 2016 | 7.47 | 12.9 | 774 | -10.0 | -66.6 | 98.7 | 21.9 | 44.6 | 3.2 | 77.4 | 84.5 | 294 | 8.29 | 13.89 | 3.37 | 26.83 | 9.97 | 96.26 | 754.70 |
| December 6, 2016 | 7.49 | 12.9 | 772 | -10.0 | -66.1 | 92.6 | 20.8 | 43.3 | 3.0 | 73.7 | 76.9 | 282 | 0.44 | 0.10 | 0.00 | 8.10 | 10.93 | 131.60 | 428.30 |
| January 27, 2017 | 7.41 | 12.9 | 775 | - | - | 88.0 | 20.3 | 37.2 | 2.9 | 58.4 | 70.3 | 284 | 0.49 | 0.17 | 0.06 | 39.91 | 11.20 | 137.10 | 348.10 |
| February 23, 2017 | 7.43 | 13 | 772 | - | - | 103.4 | 22.5 | 47.3 | 3.3 | 68.2 | 83.0 | 336 | 0.65 | 0.15 | 0.05 | 11.61 | 7.88 | 98.07 | - |
| March 23, 2017 | 7.446 | 12.9 | 759 | - | - | 98.7 | 21.4 | 42.4 | 3.6 | 72.3 | 94.5 | 282 | 0.46 | 2.98 | 0.14 | 13.19 | 7.11 | 90.98 | - |
|  |  |  |  |  |  |  |  |  |  |  |  |  |  |  |  |  |  |  |  |
| S 4 |  |  |  |  |  |  |  |  |  |  |  |  |  |  |  |  |  |  |  |
| Date | pH | T (°C) | EC (µS/cm) | δ^18^O | δD | Ca (ppm) | Mg (ppm) | Na (ppm) | K(ppm) | Cl (ppm) | SO_4_ (ppm) | HCO_3_ | As (ppb) | V (ppb) | Cr (ppb) | Fe (ppb) | Li (ppb) | B (ppb) | Sr (ppb) |
| November 5, 2014 | 7.28 | 12.4 | 655 | -10.4 | -69.6 | 116.7 | 25.1 | 6.4 | 1.6 | 9.0 | 40.2 | 433 | 1.31 | 0.74 | 9.10 | 160.10 | 1.50 | 11.74 | 413.40 |
| March 12, 2015 | - | 12.5 | 680 | -10.4 | -69.1 | 100.2 | 24.1 | 6.0 | 1.5 | 9.4 | 44.0 | 371 | 1.09 | 0.46 | 0.00 | 97.89 | 2.35 | 11.88 | 231.80 |
| December 17, 2015 | 6.98 | 13.7 | 846 | -10.2 | -67.9 | 137.7 | 30.5 | 10.8 | 2.6 | 12.9 | 81.5 | 480 | 1.78 | 0.51 | 0.06 | 204.60 | 35.18 | 167.30 | 459.00 |
| January 21, 2016 | 7.36 | 13.5 | 830 | -10.3 | -69.2 | 127.5 | 29.2 | 10.7 | 2.7 | 15.9 | 87.0 | 430 | 2.21 | 0.63 | 0.20 | 186.90 | 27.27 | 143.40 | 396.00 |
| February 26, 2016 | 7.56 | 13.5 | 814 | -10.2 | -68.5 | 131.6 | 30.5 | 11.5 | 2.4 | 15.7 | 81.8 | 458 | 1.77 | 0.57 | 0.13 | 132.70 | 25.09 | 130.40 | 353.00 |
| March 21, 2016 | 7.17 | 13.5 | 801 | -10.2 | -68.1 | 137.1 | 30.5 | 12.5 | 2.8 | 15.7 | 86.7 | 471 | 1.80 | 0.67 | 0.15 | 105.90 | 22.98 | 121.70 | 317.20 |
| April 21, 2016 | 6.88 | 13.4 | 788 | -10.3 | -68.4 | 126.3 | 29.2 | 10.9 | 3.2 | 14.6 | 71.6 | 449 | 7.56 | 8.39 | 0.27 | 42.15 | 24.38 | 121.20 | 497.10 |
| May 24, 2016 | 7.00 | 13.3 | 779 | -10.3 | -68.1 | 128.9 | 28.2 | 9.2 | 2.3 | 14.4 | 71.7 | 446 | 10.96 | 13.84 | 0.35 | 32.13 | 22.62 | 108.30 | 451.10 |
| June 23, 2016 | 7.23 | 13.6 | 776 | -10.3 | -68.8 | 104.4 | 28.7 | 9.0 | 2.5 | 14.2 | 68.1 | 379 | 12.60 | 13.65 | 0.77 | 57.39 | 22.89 | 124.30 | 508.30 |
| July 26, 2016 | 7.27 | 13.7 | 768 | -10.3 | -68.1 | 125.7 | 29.5 | 9.6 | 3.3 | 14.7 | 67.0 | 452 | 6.95 | 7.25 | 0.65 | 30.31 | 24.42 | 131.40 | 528.50 |
| August 29, 2016 | - | 13.5 | 778 | -10.3 | -68.7 | 116.7 | 26.8 | 8.4 | 2.3 | 13.0 | 70.4 | 405 | 17.50 | 22.87 | 2.50 | 63.04 | 26.09 | 146.80 | 560.30 |
| September 23, 2016 | - | - | - | - | - | - | - | - | - | - | - | - | - | - | - | - | - | - | - |
| October 27, 2016 | 7.21 | 13.3 | 775 | -10.3 | -68.4 | 127.4 | 28.8 | 9.7 | 2.7 | 14.2 | 67.7 | 452 | 7.05 | 8.22 | 3.28 | 25.36 | 24.37 | 120.30 | 482.10 |
| November 3, 2016 | 7.22 | 13.4 | 782 | -10.3 | -68.9 | 127.8 | 28.7 | 9.8 | 2.7 | 15.3 | 78.2 | 438 | 10.49 | 13.38 | 5.66 | 38.34 | 29.05 | 133.80 | 582.00 |
| December 6, 2016 | 7.26 | 13.3 | 768 | -10.3 | -68.8 | 37.8 | 14.2 | 2.3 | 0.4 | 16.3 | 73.8 | 72 | 1.60 | 0.27 | 0.02 | 41.13 | 25.87 | 151.90 | 246.60 |
| January 27, 2017 | 7.16 | 12.7 | 760 | - | - | 108.4 | 26.5 | 7.7 | 2.3 | 15.0 | 81.8 | 358 | 2.15 | 0.38 | 0.05 | 59.30 | 25.27 | 149.10 | 199.50 |
| February 23, 2017 | 7.15 | 13.2 | 755 | - | - | 122.2 | 28.2 | 9.4 | 2.5 | 12.9 | 65.6 | 437 | 1.67 | 0.34 | 0.06 | 66.62 | 16.42 | 100.10 | - |
| March 23, 2017 | 7.135 | 13.2 | 753 | - | - | 123.8 | 28.4 | 8.9 | 2.6 | 14.4 | 77.8 | 424 | 2.37 | 0.31 | 0.03 | 44.91 | 15.78 | 103.90 | - |
|  |  |  |  |  |  |  |  |  |  |  |  |  |  |  |  |  |  |  |  |
| S 5 |  |  |  |  |  |  |  |  |  |  |  |  |  |  |  |  |  |  |  |
| Date | pH | T (°C) | EC (µS/cm) | δ^18^O | δD | Ca (ppm) | Mg (ppm) | Na (ppm) | K(ppm) | Cl (ppm) | SO_4_ (ppm) | HCO_3_ | As (ppb) | V (ppb) | Cr (ppb) | Fe (ppb) | Li (ppb) | B (ppb) | Sr (ppb) |
| February 26, 2016 | 8.55 | 13.6 | 439 | -10.2 | -69.0 | 56.5 | 20.3 | 5.7 | 1.1 | 8.8 | 6.9 | 256 | 0.47 | 1.62 | 1.18 | 11.45 | 0.62 | 6.95 | 83.12 |
| March 21, 2016 | 8.34 | 13.7 | 441 | -10.2 | -68.9 | 59.2 | 20.7 | 5.8 | 1.1 | 9.0 | 7.3 | 266 | 0.29 | 1.26 | 0.86 | 10.69 | 0.39 | 4.13 | 55.26 |
| April 21, 2016 | 9.46 | 13.9 | 438 | -10.2 | -68.5 | 60.9 | 20.4 | 5.2 | 1.0 | 8.7 | 5.6 | 271 | 6.87 | 10.21 | 1.19 | 22.50 | 0.80 | 5.54 | 78.09 |
| May 24, 2016 | 8.04 | 13.7 | 440 | -10.2 | -69.9 | 60.3 | 20.3 | 4.8 | 1.3 | 10.6 | 8.3 | 258 | 9.08 | 13.81 | 1.25 | 20.47 | 0.74 | 4.58 | 75.49 |
| June 23, 2016 | 7.77 | 14.1 | 443 | -10.2 | -69.6 | 58.9 | 20.5 | 4.5 | 1.0 | 10.4 | 8.1 | 257 | 10.83 | 14.99 | 1.56 | 40.16 | 0.64 | 8.27 | 87.14 |
| July 26, 2016 | 7.79 | 14.1 | 446 | -10.2 | -69.5 | 60.8 | 21.3 | 4.6 | 1.1 | 10.4 | 8.5 | 266 | 11.01 | 16.60 | 1.73 | 35.84 | 0.64 | 9.36 | 92.03 |
| August 29, 2016 | - | 14 | 441 | -10.2 | -69.0 | 58.1 | 20.0 | 4.4 | 1.1 | 10.5 | 8.9 | 250 | 12.96 | 20.10 | 2.53 | 38.97 | 0.64 | 10.20 | 95.11 |
| September 23, 2016 | 7.84 | 13.8 | 444 | -10.2 | -69.2 | 57.2 | 19.7 | 4.4 | 1.0 | 8.8 | 7.5 | 252 | 16.76 | 28.50 | 3.44 | 32.25 | 0.59 | 9.81 | 92.68 |
| October 27, 2016 | 7.82 | 13.7 | 446 | -10.2 | -68.9 | 62.2 | 20.7 | 4.9 | 1.0 | 10.2 | 8.6 | 268 | 5.84 | 9.26 | 2.55 | 14.36 | 0.68 | 5.63 | 79.84 |
| November 3, 2016 | 7.90 | 13.6 | 445 | -10.2 | -69.2 | 60.9 | 20.6 | 4.8 | 1.0 | 10.0 | 8.4 | 266 | 9.22 | 15.31 | 3.75 | 18.25 | 1.23 | 6.42 | 93.29 |
| December 6, 2016 | 7.85 | 13.5 | 446 | -10.2 | -69.0 | 57.3 | 20.1 | 4.8 | 1.0 | 11.0 | 9.4 | 244 | 0.42 | 1.11 | 0.77 | 2.75 | 0.62 | 8.74 | 41.34 |
| January 27, 2017 | 7.70 | 13.4 | 448 | - | - | 55.2 | 19.9 | 4.6 | 1.7 | 11.3 | 8.4 | 238 | 0.53 | 1.42 | 1.09 | 2.38 | 0.59 | 8.01 | 33.76 |
| February 23, 2017 | 7.72 | 13.7 | 447 | - | - | 62.4 | 21.2 | 5.3 | 1.3 | 11.5 | 9.0 | 266 | 0.47 | 1.22 | 0.95 | 2.87 | 0.43 | 0.00 | - |
| March 23, 2017 | 7.738 | 13.7 | 449 | - | - | 61.6 | 20.9 | 4.9 | 1.1 | 11.9 | 10.9 | 257 | 0.62 | 1.22 | 0.89 | 2.80 | 0.41 | 0.00 | - |
|  |  |  |  |  |  |  |  |  |  |  |  |  |  |  |  |  |  |  |  |
| S 6 |  |  |  |  |  |  |  |  |  |  |  |  |  |  |  |  |  |  |  |
| Date | pH | T (°C) | EC (µS/cm) | δ^18^O | δD | Ca (ppm) | Mg (ppm) | Na (ppm) | K(ppm) | Cl (ppm) | SO_4_ (ppm) | HCO_3_ | As (ppb) | V (ppb) | Cr (ppb) | Fe (ppb) | Li (ppb) | B (ppb) | Sr (ppb) |
| February 26, 2016 | 8.47 | 11.7 | 359 | -11.1 | -73.6 | 62.5 | 10.0 | 4.7 | 1.0 | 7.1 | 7.3 | 228 | 0.26 | 1.05 | 0.65 | 21.00 | 0.44 | 7.55 | 71.62 |
| March 21, 2016 | 9.44 | 11.8 | 359 | -11.1 | -73.0 | 59.3 | 9.0 | 4.6 | 1.3 | 7.7 | 7.7 | 211 | 0.14 | 0.65 | 0.42 | 13.48 | 0.43 | 4.69 | 46.48 |
| April 21, 2016 | 9.02 | 12.2 | 358 | -11.1 | -73.6 | 60.7 | 9.0 | 4.9 | 0.8 | 6.0 | 6.0 | 222 | 4.88 | 7.23 | 0.54 | 21.48 | 0.52 | 4.86 | 69.04 |
| May 24, 2016 | 8.50 | 12.1 | 360 | -11.1 | -74.4 | 60.9 | 8.9 | 3.6 | 1.0 | 8.9 | 7.8 | 211 | 8.93 | 13.37 | 0.69 | 21.38 | 0.53 | 4.11 | 65.53 |
| June 23, 2016 | 7.96 | 12.9 | 366 | -11.1 | -73.7 | 61.9 | 9.3 | 3.7 | 0.8 | 8.4 | 7.1 | 219 | 8.70 | 11.51 | 0.84 | 34.86 | 0.44 | 8.41 | 78.84 |
| July 26, 2016 | 7.79 | 13.2 | 367 | -11.0 | -73.1 | 63.4 | 9.6 | 3.8 | 1.0 | 8.8 | 7.2 | 223 | 6.55 | 9.09 | 0.73 | 30.40 | 0.42 | 8.77 | 83.16 |
| August 29, 2016 | - | 12.9 | 375 | -11.0 | -72.6 | 59.9 | 9.1 | 3.6 | 0.8 | 8.1 | 7.4 | 210 | 18.15 | 26.54 | 2.02 | 44.52 | 0.47 | 10.10 | 86.15 |
| September 23, 2016 | 7.80 | 12.4 | 379 | -11.0 | -73.3 | 60.2 | 9.2 | 3.6 | 0.8 | 7.4 | 6.8 | 214 | 15.84 | 24.79 | 2.46 | 22.10 | 0.39 | 9.56 | 82.12 |
| October 27, 2016 | 7.83 | 12 | 387 | -11.0 | -72.8 | 63.8 | 10.2 | 4.0 | 0.8 | 8.7 | 7.9 | 226 | 7.66 | 11.37 | 2.06 | 16.20 | 0.53 | 5.28 | 75.67 |
| November 3, 2016 | 7.96 | 12 | 386 | -11.0 | -73.0 | 66.6 | 10.0 | 4.2 | 0.9 | 8.3 | 7.4 | 236 | 8.27 | 12.99 | 2.88 | 17.51 | 0.86 | 5.58 | 83.64 |
| December 6, 2016 | 7.77 | 11.5 | 392 | -11.0 | -72.4 | 62.5 | 9.7 | 4.3 | 0.8 | 8.0 | 7.9 | 222 | 0.17 | 0.55 | 0.39 | 0.00 | 0.46 | 9.99 | 42.21 |
| January 27, 2017 | 7.67 | 10.6 | 405 | - | - | 59.8 | 9.5 | 3.6 | 0.8 | 9.2 | 8.9 | 205 | 0.31 | 0.77 | 0.65 | 3.78 | 0.42 | 8.57 | 32.68 |
| February 23, 2017 | 7.69 | 11.3 | 401 | - | - | 68.4 | 10.3 | 4.9 | 0.9 | 8.9 | 9.3 | 240 | 0.21 | 0.63 | 0.49 | 3.90 | 0.31 | 0.00 | - |
| March 23, 2017 | 7.752 | 12.1 | 400 | - | - | 68.2 | 10.3 | 4.5 | 1.2 | 9.8 | 11.6 | 233 | 0.24 | 0.64 | 0.52 | 4.13 | 0.29 | 0.00 | - |
|  |  |  |  |  |  |  |  |  |  |  |  |  |  |  |  |  |  |  |  |
| S 7 |  |  |  |  |  |  |  |  |  |  |  |  |  |  |  |  |  |  |  |
| Date | pH | T (°C) | EC (µS/cm) | δ^18^O | δD | Ca (ppm) | Mg (ppm) | Na (ppm) | K(ppm) | Cl (ppm) | SO_4_ (ppm) | HCO_3_ | As (ppb) | V (ppb) | Cr (ppb) | Fe (ppb) | Li (ppb) | B (ppb) | Sr (ppb) |
| February 26, 2016 | 8.46 | 9.9 | 446 | -10.2 | -69.6 | 78.1 | 10.9 | 5.0 | 1.6 | 6.9 | 9.4 | 282 | 0.31 | 0.44 | 0.25 | 47.00 | 1.06 | 13.79 | 91.81 |
| March 21, 2016 | 9.48 | 10.5 | 452 | -10.3 | -68.0 | 70.8 | 9.0 | 4.3 | 1.2 | 8.2 | 12.2 | 242 | 0.21 | 0.38 | 0.24 | 74.43 | 0.79 | 10.35 | 73.63 |
| April 21, 2016 | 8.55 | 9.9 | 453 | -10.3 | -68.6 | 82.2 | 9.5 | 5.9 | 1.3 | 7.6 | 10.6 | 287 | 6.49 | 8.90 | 0.36 | 100.70 | 1.10 | 9.35 | 138.50 |
| May 24, 2016 | 7.68 | 11.2 | 462 | -10.2 | -68.7 | 84.0 | 10.3 | 4.1 | 1.3 | 6.4 | 8.5 | 297 | 9.61 | 13.43 | 0.48 | 53.82 | 1.16 | 9.34 | 137.30 |
| June 23, 2016 | 7.77 | 13.5 | 446 | -10.3 | -68.8 | 78.0 | 10.4 | 3.9 | 1.4 | 7.3 | 9.4 | 275 | 13.97 | 18.56 | 0.86 | 49.28 | 1.05 | 14.40 | 145.00 |
| July 26, 2016 | 7.82 | 14.8 | 400 | -10.3 | -68.0 | 67.1 | 10.0 | 4.1 | 2.7 | 9.0 | 8.0 | 241 | 10.65 | 15.26 | 0.66 | 79.69 | 0.97 | 13.30 | 123.80 |
| August 29, 2016 | - | 14.5 | 375 | -10.4 | -69.5 | 62.3 | 10.3 | 3.7 | 1.6 | 7.7 | 8.1 | 227 | 15.36 | 23.57 | 1.51 | 98.15 | 0.88 | 12.84 | 115.10 |
| September 23, 2016 | 7.60 | 14.3 | 468 | -10.2 | -68.0 | 79.3 | 10.2 | 3.7 | 1.7 | 6.3 | 8.5 | 283 | 14.23 | 22.70 | 2.29 | 111.80 | 1.12 | 18.37 | 157.10 |
| October 27, 2016 | 7.67 | 13.1 | 438 | -10.0 | -65.8 | 72.0 | 9.1 | 3.8 | 2.3 | 6.6 | 8.6 | 255 | 7.42 | 10.27 | 1.89 | 117.80 | 1.10 | 13.45 | 131.00 |
| November 3, 2016 | 7.71 | 10.6 | 456 | -10.0 | -67.0 | 85.3 | 10.3 | 4.0 | 1.4 | 6.2 | 8.1 | 302 | 9.50 | 13.89 | 2.82 | 159.80 | 1.44 | 12.08 | 152.80 |
| December 6, 2016 | 7.75 | 6.8 | 463 | -10.1 | -67.1 | 77.1 | 10.1 | 4.0 | 1.0 | 6.3 | 11.0 | 270 | 0.39 | 0.27 | 0.17 | 297.70 | 1.19 | 16.61 | 79.62 |
| January 27, 2017 | 7.53 | 6.2 | 438 | - | - | 71.5 | 9.6 | 3.4 | 1.7 | 7.9 | 10.8 | 247 | 0.66 | 0.32 | 0.29 | 532.60 | 1.02 | 14.39 | 61.11 |
| February 23, 2017 | 7.55 | 7.5 | 446 | - | - | 27.9 | 3.6 | 1.8 | 1.0 | 8.6 | 18.0 | 67 | 0.50 | 0.25 | 0.16 | 378.80 | 0.72 | 0.00 | - |
| March 23, 2017 | 7.752 | 12.1 | 400 | - | - | 84.3 | 11.1 | 4.3 | 1.4 | 9.7 | 24.2 | 277 | 0.41 | 0.18 | 0.07 | 228.40 | 0.68 | 1.82 | - |
|  |  |  |  |  |  |  |  |  |  |  |  |  |  |  |  |  |  |  |  |
| S 8 |  |  |  |  |  |  |  |  |  |  |  |  |  |  |  |  |  |  |  |
| Date | pH | T (°C) | EC (µS/cm) | δ^18^O | δD | Ca (ppm) | Mg (ppm) | Na (ppm) | K(ppm) | Cl (ppm) | SO_4_ (ppm) | HCO_3_ | As (ppb) | V (ppb) | Cr (ppb) | Fe (ppb) | Li (ppb) | B (ppb) | Sr (ppb) |
| June 15, 2016 |  |  |  | - | - | 50.1 | 5.8 | 1.4 | 0.4 | 3.2 | 23.8 | 149.58 | 0.70 | 0.84 | 0.66 | 18.32 | 0.91 | 4.10 | 240.00 |
| September 27, 2016 |  |  |  | -10.6 | -70.2 | 48.6 | 5.9 | 1.3 | 0.4 | 2.6 | 22.0 | 148.74 | 1.82 | 3.05 | 0.90 | 19.34 | 0.90 | 4.53 | 253.70 |
| October 25, 2016 |  |  |  | -10.6 | -70.3 | 48.9 | 6.5 | 1.5 | 0.7 | 2.8 | 25.5 | 148.78 | 0.10 | 0.27 | 0.13 |  | 0.80 | 7.11 | 280.70 |
| November 4, 2016 |  |  |  | -10.6 | -70.8 | 50.6 | 6.4 | 1.4 | 0.6 | 2.5 | 24.2 | 155.42 | 6.91 | 13.27 | 1.37 | 15.62 | 1.09 | 0.34 | 260.50 |
| November 11, 2016 |  |  |  | -10.6 | -69.8 | 47.1 | 6.2 | 1.4 | 0.4 | 3.9 | 25.6 | 138.61 | 0.16 | 0.23 | 0.21 |  | 1.02 | 5.81 | 306.50 |
| November 23, 2016 |  |  |  | -10.6 | -70.0 | 46.4 | 6.2 | 1.4 | 0.4 | 2.6 | 28.1 | 136.02 | 0.13 | 0.26 | 0.18 | 11.69 | 1.18 | 4.14 | 265.40 |
| December 14, 2016 |  |  |  | -10.6 | -70.0 | 48.2 | 6.4 | 1.5 | 0.5 | 2.7 | 28.5 | 141.92 | 0.07 | 0.16 | 0.08 | 3.06 | 0.96 | 4.97 | 128.20 |
| January 12, 2017 |  |  |  | -10.6 | -69.9 | 44.1 | 5.1 | 1.1 | 0.5 | 2.2 | 18.6 | 135.23 | 0.14 | 0.39 | 0.26 | 8.29 | 0.98 | 2.95 | 93.26 |
| February 8, 2017 |  |  |  | - | - | 46.0 | 5.4 | 1.3 | 0.4 | 4.0 | 21.8 | 135.82 | 0.14 | 0.15 | 0.17 | 9.48 | 0.61 | 0.00 | - |
| March 23, 2017 |  |  |  | - | - | - | - | - | - | - | - | - | - | - | - | - | - | - | - |
|  |  |  |  |  |  |  |  |  |  |  |  |  |  |  |  |  |  |  |  |
| W |  |  |  |  |  |  |  |  |  |  |  |  |  |  |  |  |  |  |  |
| Date | pH | T (°C) | EC (µS/cm) | δ^18^O | δD | Ca (ppm) | Mg (ppm) | Na (ppm) | K(ppm) | Cl (ppm) | SO_4_ (ppm) | HCO_3_ | As (ppb) | V (ppb) | Cr (ppb) | Fe (ppb) | Li (ppb) | B (ppb) | Sr (ppb) |
| October 27, 2016 | - | - |  | - | - | 87.0 | 16.6 | 15.1 | 1.5 | 22.2 | 36.3 | 303 | - | - | - | - | - | - | - |
